# What do Cochrane systematic reviews say about the clinical effectiveness of screening and diagnostic tests for cancer? O que as revisões sistemáticas de Cochrane falam sobre a eficácia clínica dos testes de rastreamento e diagnóstico para o câncer?

**DOI:** 10.1590/1516-3180.2017.0171110717

**Published:** 2017-05-29

**Authors:** André Tito Pereira Bueno, Vladimir Lisboa Capelasso, Rafael Leite Pacheco, Carolina de Oliveira Cruz Latorraca, Tiago Biachi de Castria, Daniela Vianna Pachito, Rachel Riera

**Affiliations:** I Undergraduate Medical Student, Escola Paulista de Medicina (EPM), Universidade Federal de São Paulo (Unifesp), São Paulo (SP), Brazil.; II MSc. Psychologist, Evidence-Based Health Program, Universidade Federal de São Paulo (Unifesp), São Paulo (SP), Brazil; and Assistant Researcher, Cochrane Brazil, São Paulo (SP), Brazil.; III MD, PhD. Clinical Oncologist, Instituto do Câncer do Estado de São Paulo, Faculdade de Medicina da Universidade Federal de São Paulo (USP) and Hospital Sírio Libanês, São Paulo (SP), Brazil.; IV MD, MSc. Neurologist and Postgraduate Student, Evidence-Based Health Program, Universidade Federal de São Paulo (Unifesp), São Paulo (SP), and Assistant Researcher, Cochrane Brazil, São Paulo (SP), Brazil.; V MD, MSc, PhD. Rheumatologist and Adjunct Professor, Discipline of Evidence-Based Medicine, Escola Paulista de Medicina (EPM), Universidade Federal de São Paulo (Unifesp), São Paulo (SP), Brazil.

**Keywords:** Diagnosis, Early detection of cancer, Treatment outcome, Review [publication type], Evidence-based practice

## Abstract

**CONTEXT AND OBJECTIVE:**

The purpose of screening tests for cancer is to detect it at an early stage in order to increase the chances of treatment. However, their unrestrained use may lead to unnecessary examinations, overdiagnosis and higher costs. It is thus necessary to evaluate their clinical effects in terms of benefits and harm.

**DESIGN AND SETTING:**

Review of Cochrane systematic reviews, carried out in the Discipline of Evidence-Based Medicine, Escola Paulista de Medicina, Universidade Federal de São Paulo.

**METHODS:**

Cochrane reviews on the clinical effectiveness of cancer screening procedures were included. Study titles and abstracts were independently assessed by two authors. Conflicts were resolved by another two authors. Findings were summarized and discussed.

**RESULTS:**

Seventeen reviews were selected: fifteen on screening for specific cancers (bladder, breast, colorectal, hepatic, lung, nasopharyngeal, esophageal, oral, prostate, testicular and uterine) and two others on cancer in general. The quality of evidence of the findings varied among the reviews. Only two reviews resulted in high-quality evidence: screening using low-dose computed tomography scans for high-risk individuals seems to reduce lung cancer mortality; and screening using flexible sigmoidoscopy and fecal occult blood tests seems to reduce colorectal cancer mortality.

**CONCLUSION:**

The evidence found through Cochrane reviews did not support most of the commonly used screening tests for cancer. It is recommended that patients should be informed of the possibilities of false positives and false negatives before they undergo the tests. Further studies to fully assess the effectiveness of cancer screening tests and adverse outcomes are required.

## RESUMO

**CONTEXTO E OBJETIVO:**

O objetivo do teste de rastreamento para o câncer é detectá-lo em um estágio inicial, a fim de aumentar as chances de cura. Contudo, seu uso descomedido pode levar a exames desnecessários, sobrediagnóstico e aumento de custos. Portanto, é necessário que se avalie a repercussão clínica do rastreamento em termos de benefícios e riscos.

**TIPO DE ESTUDO E LOCAL:**

Revisão de revisões sistemáticas Cochrane realizada na Disciplina de Medicina Baseada em Evidências da Escola Paulista de Medicina (EPM), Universidade Federal de São Paulo (UNIFESP).

**MÉTODOS:**

Foram incluídas revisões sobre efetividade clínica de testes de rastreamento para câncer. Os títulos e resumos foram avaliados independentemente por dois autores e divergências foram resolvidas por outros dois. Os achados foram resumidos e discutidos.

R**ESULTADOS:** 17 revisões sistemáticas foram incluídas: 15 sobre rastreamento para cânceres específicos (vesical, mamário, colorretal, hepático, pulmonar, nasofaríngeo, esofágico, oral, prostático, testicular, uterino) e duas para câncer em geral. A qualidade das evidências encontradas pelas revisões variou muito. Duas revisões encontraram evidências de alta qualidade: o rastreamento com tomografia em dose baixa em pacientes de alto risco parece reduzir a mortalidade por câncer pulmonar; e rastreamento com sigmoidoscopia flexível e pesquisa de sangue oculto nas fezes parece reduzir a mortalidade por câncer colorretal.

**CONCLUSÃO:**

As evidências de revisões sistemáticas Cochrane não indicam a realização dos testes mais usados para rastreamento de câncer. Recomenda-se que os pacientes sejam informados sobre as possibilidades de falsos positivos e de falsos negativos antes de serem submetidos aos testes. Estudos adicionais para avaliar melhor a eficácia de testes de rastreamento para o câncer e os eventos adversos são necessários.

## INTRODUCTION

Cancer is one of the leading causes of morbidity and mortality worldwide and was responsible for 8.8 million deaths in 2015. Its incidence is expected to increase by about 70% over the next two decades.^1^ Its vast importance can be perceived from its economic impact on public health, which was estimated as US$ 1.16 trillion in 2010.^2^

Screening tests are one of the main pillars of early detection. They are performed on healthy individuals presenting different levels of risk, with the aim of detecting the disease at an early stage, even before any symptom becomes noticeable. However, carrying out these tests within screening-based programs is only justified if they lead to better health outcomes than would be achieved by treating diseases at a later stage.^3^ Theoretically, the aim in performing such tests is to minimize the number of people with the disease who pass through them undetected (high sensitivity), while also minimizing the number of people without cancer who are selected for further examination (high specificity).^4^

The first concept that needs to be underscored in making a decision to implement screening is overdiagnosis. This is epidemiologically defined as a situation of diagnosing a condition that during the patient’s lifetime would have remained indolent if left undetected.^5^ The main consequence from this would be overtreatment, which would have no benefits for the patients, might cause harm and possibly would generate costs. Additionally, it could divert healthcare professionals and resources away from the most severely ill patients.^5^ One well-known example of overdiagnosis took place during the 1950s in the United States, when breast self-examination (BSE) was widely advocated, only for it to be concluded in the 1990s that BSE had no impact on reducing breast cancer mortality.^6^

A second important concept to have in mind is the likelihood of false-negative results. Aside from the delay in detection of the disease and its further development, such situations could lead to legal action from patients affected by them, and more importantly, could reduce public confidence in screening policies.^7^

Another important aspect of screening programs to be taken into consideration is that the likelihood that a positive test will give a correct result (positive predictive value) is strongly dependent on the prevalence of the disease within the population. Hence, the effectiveness of screening programs varies between different regions. Furthermore, the effectiveness of screening tests is also affected by other variables such as adequate infrastructure, resources and professional qualification.^4^ For example, although magnetic resonance imaging may have higher efficacy for detection of breast cancer, compared with conventional mammography, the infrastructure cost and the high number of false-positive results considerably increases its costs and harm, thus leading to a preference for conventional mammography.^8^

Finally, screening strategies should be implemented only in settings in which further investigation and treatment are warranted for all individuals, whenever necessary. Otherwise, there is no benefit even for evidence-based screening protocols.

## OBJECTIVE

The objective of this study was to summarize the evidence from Cochrane systematic reviews regarding the clinical effectiveness of screening tests for detection of different types of cancer.

## METHODS

### Design and setting

This was a review of Cochrane systematic reviews carried out in the Discipline of Evidence-Based Medicine, Escola Paulista de Medicina - Universidade Federal de São Paulo (EPM-Unifesp). This article was specifically developed for the section “Cochrane Highlights”, which is an initiative for disseminating Cochrane reviews. This initiative resulted from a formal partnership between the São Paulo Medical Journal and the Cochrane Collaboration, and it is supported by Cochrane Brazil.

### Criteria for including reviews

- Types of studies Only Cochrane systematic reviews on effectiveness and safety, including randomized, quasi-randomized or non-randomized clinical trials as primary studies, were used in producing the present review. Systematic reviews focusing on ­diagnostic accuracy were excluded. Protocols, withdrawn reviews and previous versions of updated reviews were not taken into consideration.
- Types of participants
- All types of participants, regardless of sex and age or other characteristics (i.e. different risks for cancer, genetic factors, previous cancer), were included.
- Types of intervention Any screening approaches for cancer detection were included.
- Type of outcomes

Clinical outcomes involving morbidity and mortality were considered.

### Search for reviews

We developed and applied a systematic search strategy in the Cochrane Library on December 19, 2016 (**Table 1**). Two researchers (ATPB and VLC) selected and assessed systematic reviews with themes that showed a correlation with the goals of this review. A third researcher (RR or RLP) resolved any conflicts that arose, when necessary.

**Table 1:**
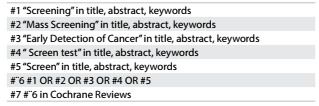
Search strategy in Cochrane Library. December 19, 2016

## RESULTS

### Search results

The search resulted in 927 Cochrane systematic reviews. After the titles and abstracts had been screened, 17 reviews were found to be related to the theme and fulfilled our inclusion criteria.^9^^,^^10^^,^^11^^,^^12^^,^^13^^,^^14^^,^^15^^,^^16^^,^^17^^,^^18^^,^^19^^,^^20^^,^^21^^,^^22^^,^^23^^,^^24^^,^^25^ Among these, 7 reviews assessed a screening method only for the general population, 7 only for a specific subpopulation and 3 for both. The reviews assessed screening approaches for the following types of cancer: cancer in general (n = 2), ­bladder (n = 1), breast (n = 4), colorectal (n = 2), hepatic (n = 1), lung (n = 1), nasopharyngeal (n = 1), esophageal (n = 1), oral (n = 1), prostate (n = 1), testicular (n = 1) and uterine (n = 1).

### Results from systematic reviews

The 17 systematic reviews are presented below. Additionally, for those that included at least one primary study, a brief summary is then presented. The issues addressed, the main findings from each screening approach and the quality of the evidence (based on the GRADE approach^26^) are presented in **Table 2**.

**Table 2:**
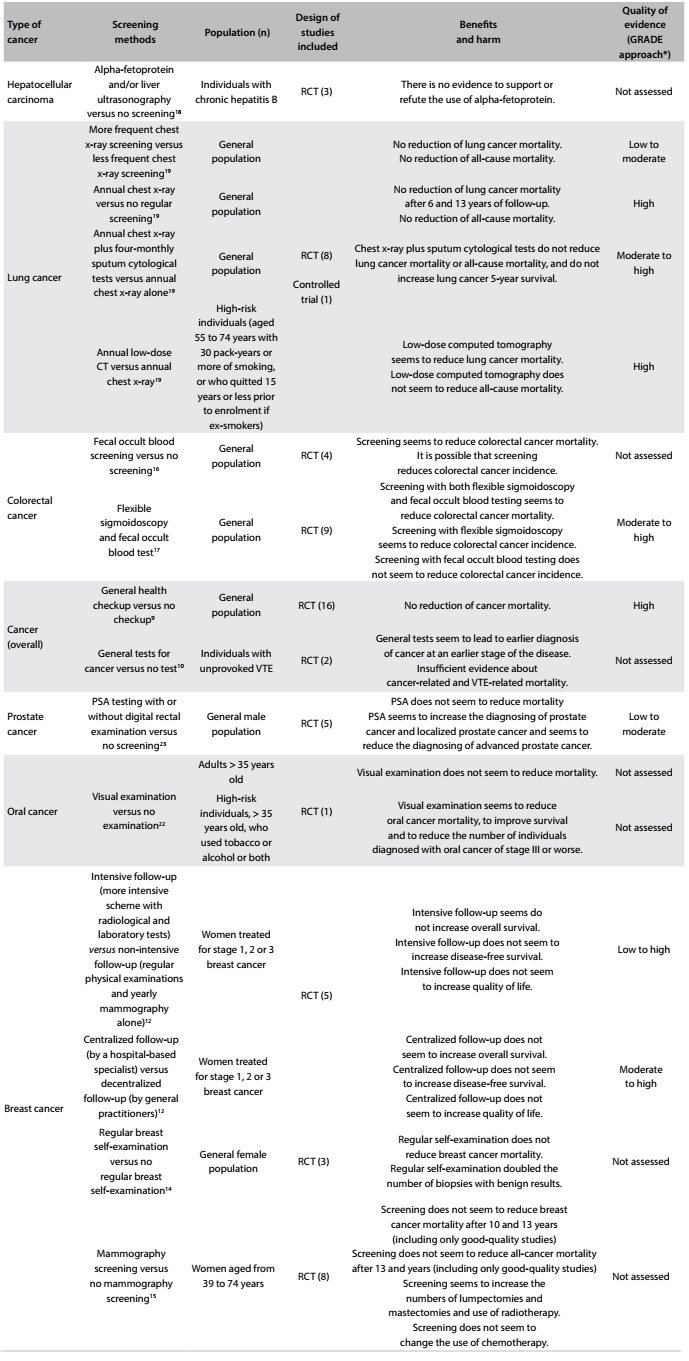
Issues addressed, main findings and quality of evidence from systematic reviews that included at least one randomized clinical trial

RCT = randomized controlled trial; *GRADE = Grading of Recommendations Assessment, Development and Evaluation;^26^ VTE = venous thromboembolism; PSA = prostate-specific antigen*.*

#### General health checkup among adults

The purpose of general health checkups is to detect disease and risk factors for disease with the aim of reducing morbidity and mortality. In this review,^9^ the authors aimed to quantify the benefits and harm of general health checkups with an emphasis on patient-relevant outcomes such as morbidity and mortality. The results showed that there was no difference between the checkup and ‘no checkup’ groups regarding cancer-related mortality (risk ratio [RR] 1.02; 95% confidence interval [95% CI] 0.92 to 1.12; eight randomized clinical trials [RCTs]; 139,290 participants; 3663 deaths). The authors concluded that general health checkups did not reduce cancer-related morbidity or mortality, but that the number of new diagnoses increased. Important harmful outcomes, such as the number of follow-up diagnostic procedures or short term psychological effects, were often not studied or reported and many trials had methodological problems. With the large number of participants and deaths included and the long follow-up periods used, and considering that cancer mortality was not reduced, general health checkups are unlikely to be beneficial. For further details, refer to the original abstract, available at: http://onlinelibrary.wiley.com/doi/10.1002/14651858.CD009009.pub2/full.

#### Tests for cancer in patients with unprovoked venous thromboembolism

An unprovoked venous thromboembolism (VTE) is one that affects patients with no underlying or immediately predisposing risk factors. It can frequently be the first clinical manifestation of an underlying malignancy. This raises the question of whether patients with an unprovoked VTE should be investigated for an underlying cancer. The treatment for VTE differs between cancer and non-cancer patients, and a correct diagnosis would ensure that patients received optimal treatment for VTE, to prevent recurrence and further morbidity. The objectives of this review^10^ were to determine whether cancer testing for patients with a first episode of unprovoked VTE is effective in reducing cancer and VTE-related mortality and morbidity and to establish which tests for cancer are most useful. Two RCTs (396 patients) assessed the effect of routine cancer tests versus clinically indicated cancer tests for patients with an unprovoked VTE. Cancer-related mortality did not differ between the two testing approaches (odds ratio [OR] 0.49; 95% CI 0.15 to 1.67; P = 0.26; moderate quality evidence). Neither of the studies measured all-cause mortality, VTE-related morbidity and mortality, adverse events, or patient satisfaction. The authors concluded that there was currently insufficient evidence for firm conclusions regarding the effectiveness of cancer testing for a first episode of unprovoked VTE. The results were imprecise and could be consistent with either harm or benefit. Further good-quality RCTs are needed before definitive conclusions can be reached. For further details, refer to the original abstract, available at: http://onlinelibrary.wiley.com/doi/10.1002/14651858.CD010837.pub2/full.

#### Follow-up strategies for women treated for early breast cancer 

Follow-up examinations are often used, after primary treatment for women with breast cancer, to detect recurrences at an early (asymptomatic) stage. The objective of this systematic review^12^ was to evaluate the effectiveness of different follow-up strategies for detecting distant metastasis, regarding mortality, morbidity and quality of life among women who had been treated for stage 1, 2 or 3 breast cancer. This updated review included five RCTs involving 4,023 women with breast cancer (clinical stage 1, 2 or 3). Two trials involving 2,563 women compared follow-up based on clinical visits and mammography with a more intensive scheme including radiological and laboratory tests. The data showed that there were no significant differences between the two strategies in relation to the following outcomes:

- Overall survival (hazard ratio [HR] 0.98; 95% CI 0.84 to 1.15; two RCTs; 2,563 participants; high-quality evidence);
- Disease-free survival (HR 0.84; 95% CI 0.71 to 1.00; two RCTs; 2,563 participants; low-quality evidence);
- Quality-of-life measurements (one RCT; 639 participants; high-quality evidence).

The results from subgroup analyses according to patient age, tumor size and lymph node status before primary treatment were consistent. In 1999, 10-year follow-up data became available for one RCT, and no significant differences in overall survival were found. Two RCTs compared follow-up performed by a hospital-based specialist versus follow-up performed by general practitioners and showed the following results according to outcome:

- Overall survival: no difference between the strategies (HR 1.07; 95% CI 0.64 to 1.78; one RCT; 968 participants; moderate-quality evidence);
- Time elapsed until detection of recurrence: no difference between the strategies (HR 1.06; 95% CI 0.76 to 1.47; two studies; 1,264 participants; moderate-quality evidence);
- Quality of life: no difference between the strategies (one RCT; 356 participants; high-quality evidence);
- Patient satisfaction: greater among patients treated by general practitioners.

One RCT (196 women) compared regularly scheduled follow-up visits versus less frequent visits restricted to the time of mammography. No significant differences emerged in relation to interim use of telephone contacts and frequency of consultations with general practitioners. The authors concluded that follow-up programs based on regular physical examinations and yearly mammography alone are as effective as more intensive approaches based on regularly performing laboratory and instrumental tests, in terms of the time that elapsed until detection of recurrence, overall survival and quality of life. In two RCTs, follow-up care provided by trained and untrained general practitioners working in an organized practice setting had comparable effectiveness to that delivered by hospital-based specialists, in terms of overall survival, detection of recurrence and quality of life. For further details, refer to the original abstract, available at: http://onlinelibrary.wiley.com/doi/10.1002/14651858.CD001768.pub3/full.

#### Regular self-examination or clinical examination for early detection of breast cancer

The practice of clinical breast examination and breast self-examination has been widely disseminated and advocated for years as a general screening method for diagnosing breast cancer. This systematic review^14^ aimed to assess the clinical effectiveness of these practices and found two large population-based studies (n = 388,535 women) that had been conducted in Shanghai and Russia, comparing breast self-examination with no intervention (control group). The results showed:

- There was no statistically significant difference in breast cancer mortality between the groups (RR 1.05; 95% CI 0.90 to 1.24; 587 deaths in total);
- In Russia, more cancers were diagnosed in the self-examination group than in the control group (RR 1.24; 95% CI 1.09 to 1.41), whereas in Shanghai there was no statistical difference between the groups (RR 0.97; 95% CI 0.88 to 1.06);
- In the screening groups, nearly twice as many biopsies (n = 3,406) with benign results were conducted, compared with the control group (n = 1,856) (RR 1.88; 95% CI 1.77 to 1.99).

Another large population-based study on combined breast self-examination and clinical breast examination was also included, but this study was discontinued due to lack of compliance with follow-up, and no conclusions were drawn. The authors of the systematic review concluded that there was no suggestion of any beneficial effect from breast self-examination screening. Rather, there was only increased harm due to increased numbers of benign lesions identified, with consequently increased numbers of biopsies performed. Therefore, screening by means of physical examination and breast self-examination is no longer recommended. For further details, refer to the original abstract, available at: http://onlinelibrary.wiley.com/doi/10.1002/14651858.CD003373/full.

#### Screening for breast cancer via mammography

This review^15^ aimed to assess the benefits and harm of mammographic screening for breast cancer. It included seven RCTs (600,000 women, of ages ranging from 39 to 74 years) in which results from mammographic screening and without mammographic screening were compared. The pooled results from three RCTs with good methodological quality showed that mammographic screening did not reduce:

- Breast cancer-related mortality after 13 years (RR 0.90; 95% CI 0.79 to 1.02);
- All cancer-related mortality, including breast cancer, after 10 years (RR 1.02; 95% CI 0.95 to 1.10);
- All-cause mortality after 13 years (RR 0.99; 95% CI 0.95 to 1.03).

The total numbers of lumpectomies and mastectomies were significantly larger (31% higher) in the screened groups (RR 1.31; 95% CI 1.22 to 1.42), as were the numbers of mastectomies (20% higher) (RR 1.20; 95% CI 1.08 to 1.32). The use of chemotherapy and radiotherapy was similarly increased in both groups (screened and non-screened). The authors concluded that if the overdiagnosis and overtreatment rates were 30%, 10 years of screening on 2000 women would have the outcomes that one women would avoid dying of breast cancer and 10 healthy women would be treated unnecessarily. Also, 200 women would experience significant psychological distress (anxiety and uncertainty) due to false-positive findings. Furthermore, the authors stated that given the substantial advances in treatment and the greater breast cancer awareness that had been achieved since the time when these RCTs were undertaken, it was most likely that the absolute effect of screening at the time of writing was smaller than at the time of the trials. They also noted that there were recent studies showing greater degrees of overdiagnosis and very little or no reduction in the incidence of advanced cancers through screening. For further details, refer to the original abstract, available at: http://onlinelibrary.wiley.com/doi/10.1002/14651858.CD001877.pub5/full.

#### Screening for colorectal cancer using the fecal occult blood test (Hemoccult)

The fecal occult blood test (FOBT) is used as a population-based screening for reducing mortality due to colorectal cancer. This review^16^ evaluated whether screening using FOBT (guaiac or immunochemical) indeed reduces colorectal cancer mortality and what the benefits and harm from screening might be. Meta-analyses on four RCTs showed that participants allocated to FOBT screening had a reduction in the risk of colorectal cancer mortality of 16%, compared with no FOBT screening (RR 0.84; 95% CI 0.78-0.90). The authors concluded that benefits from the screening included: moderate reduction in mortality due to colorectal cancer; possible reduction in the incidence of cancer through detection and removal of colorectal adenomas; and, potentially, the less invasive surgery that earlier treatment of colorectal cancers may involve. The harmful effects from the screening included: the psychosocial consequences of receiving a false-positive result; the potentially significant complications of colonoscopy or a false-negative result; the possibility of overdiagnosis (leading to unnecessary investigations or treatment); and the complications associated with treatment. For further details, refer to the original abstract, available at: http://onlinelibrary.wiley.com/doi/10.1002/14651858.CD001216.pub2/full.

#### Flexible sigmoidoscopy versus fecal occult blood testing for colorectal cancer screening 

Screening using FOBT and flexible sigmoidoscopy have been shown to reduce mortality due to colorectal cancer in randomized controlled trials. The objective of this review^17^ was to compare the effectiveness of screening for colorectal cancer using flexible sigmoidoscopy versus screening using the FOBT. For this purpose, a search for RCTs comparing screening using flexible sigmoidoscopy or FOBT, with each other or with no screening, was conducted. Only studies reporting mortality due to colorectal cancer were included. FOBT had to be repeated (annually or biennially).

Nine RCTs involving 338,467 individuals randomized to screening and 405,919 individuals to control groups were identified. Five RCTs compared flexible sigmoidoscopy with no screening and four studies compared repetitive guaiac-based FOBT (annually and biennially) with no screening. No studies directly comparing the two screening methods were found. Colorectal cancer mortality was lower when flexible sigmoidoscopy was used (RR 0.72; 95% CI 0.65 to 0.79; high-quality evidence) than with no screening and when FOBT was used (RR 0.86; 95% CI 0.80 to 0.92; high-quality evidence). Based on indirect comparison of the two screening methods, the RR of dying due to colorectal cancer was 0.85 (95% CI 0.72 to 1.01; low-quality evidence) for screening using flexible sigmoidoscopy, in comparison with FOBT. No complications occurred after the FOBT test itself, but 0.03% of the participants suffered a major complication after follow-up. Among more than 60,000 flexible sigmoidoscopy screening procedures and almost 6,000 work-up colonoscopies, a major complication was recorded in 0.08% of the participants. The authors concluded there was high-quality evidence showing that screening by means of flexible sigmoidoscopy and FOBT reduced the mortality due to colorectal cancer. On the other hand, there was low-quality indirect evidence that screening using one of the approaches reduced colorectal cancer deaths more than the other. Major complications associated with screening require validation from studies with more complete reporting of harm. For further details, refer to the original abstract, available at: http://onlinelibrary.wiley.com/doi/10.1002/14651858.CD009259.pub2/full.

#### Alpha-fetoprotein and/or ultrasonography for liver cancer screening in patients with hepatitis B 

This review^18^ included three RCTs, and each of them used a different method for liver cancer screening. The first one, conducted in Shanghai, China, randomized the participants to screening every six months using alpha-fetoprotein and ultrasonography (n = 9,373) versus no screening (n = 9,443). The second trial, conducted in Toronto, Canada, on 1,069 participants with chronic hepatitis B, compared screening every six months with alpha-fetoprotein alone (n = 532) versus alpha-fetoprotein and ultrasound (n = 538) over a five-year period. The last study, conducted in Taiwan, was designed as a cluster randomized trial to determine the optimal interval for screening using alpha-fetoprotein and ultrasound. Screening intervals of 4 and 12 months were compared in the groups.

The results from the first two studies did not show any significant conclusions. The third study only showed increased incidence of hepatocellular carcinoma in the four-monthly screening group.

None of the three trials included any reports on adverse events. The authors of this systematic review judged that the specificity and sensitivity of the trials were poor.

The results were inconclusive since there was not enough evidence to evaluate the value of alpha-fetoprotein or ultrasound screening, or both, for patients with hepatocellular carcinoma who were positive for hepatitis B surface antigen (HBsAg). For further details, refer to the original abstract, available at: http://onlinelibrary.wiley.com/doi/10.1002/14651858.CD002799.pub2/full.

#### Screening for lung cancer

The objective of this review^19^ was to determine whether screening for lung cancer (using sputum examinations, chest radiography or chest computed tomography) reduced mortality. Eight RCTs and one non-randomized trial (453,965 subjects) were included and these showed the following:

- Comparison of annual chest X-ray screening for smokers and non-smokers versus no screening: no reduction in lung cancer mortality (RR 0.99; 95% CI 0.91 to 1.07; one study);
- Comparison of different frequencies of chest X-ray screening: frequent screening was associated with an 11% relative increase in mortality due to lung cancer, compared with less frequent screening (RR 1.11; 95% CI 1.00 to 123; low-quality evidence);
- Comparison of screening using chest X-ray plus sputum cytological tests versus screening using chest X-ray alone: no reduction in lung cancer mortality (RR 0.88; 95% CI 0.74 to 1.03);
- Comparison of annual low-dose computed tomography screening versus annual chest X-ray screening for high-risk smokers and ex-smokers: low-dose computed tomography screening was associated with a 20% decrease in mortality due to lung cancer (RR 0.80; 95% CI 0.70 to 0.92).

The authors concluded there was no current evidence to support screening for lung cancer using chest radiography or sputum cytological tests. Annual low-dose computed tomography screening was correlated with a reduction in lung cancer mortality among high-risk smokers. However, it is essential to obtain further data on the cost-effectiveness of screening and the relative harm and benefits of screening across a range of different groups at risk and different settings. For further details, refer to the original abstract, available at: http://onlinelibrary.wiley.com/doi/10.1002/14651858.CD001991.pub3/full.

#### Screening programs in oral cancer

This review^22^ aimed to assess the effectiveness of screening (using visual examination, toluidine blue, fluorescence imaging or brush biopsy) for early detection and prevention of oral cancer. Only one RCT, with a 15-year follow-up, met the inclusion criteria. This RCT found that there was a 24% reduction in mortality associated with screening (30/100,000 person-years versus 39/100,000; RR 0.76; 95% CI 0.60 to 0.97) among high-risk individuals who used tobacco or alcohol or both. Moreover, the screening group presented a 19% reduction in the number of individuals diagnosed with oral cancer in stage 3 or higher (RR 0.81; 95% CI 0.70 to 0.93). The authors concluded that there was evidence that visual examination as part of a population-based screening program could reduce oral cancer mortality among high-risk individuals and that there was a reduction in staging and an improvement in survival across the population. There was no evidence to support use of adjunctive technologies (including toluidine blue, brush biopsy or fluorescence imaging) as a screening tool to reduce oral cancer mortality. However, the evidence was limited to one study that presented high risk of bias and which did not consider the effect of cluster randomization in the analysis. For further details, refer to the original abstract, available at: http://onlinelibrary.wiley.com/doi/10.1002/14651858.CD004150.pub4/full.

#### Screening for prostate cancer

Routine mass, selective or opportunistic screening for prostate cancer has resulted in considerable debate regarding whether this screening indeed reduces mortality and improves quality of life. The objectives of this systematic review^23^ were to assess whether screening for prostate cancer reduces prostate cancer-specific mortality or all-cause mortality and to determine its impact on quality of life and adverse events, so as to better inform decision-making relating both to individual patients and to healthcare policy.

Five RCTs (341,342 participants, aged from 45 to 80 years, with duration of follow-up from 7 to 20 years) were included and all of them involved prostate-specific antigen testing, with or without digital rectal examination. There was no significant difference in prostate cancer-specific mortality between the screened and non-screened groups (RR 1.00; 95% CI 0.86 to 1.17). Three RCTs presented high risk of bias, and the other two were classified as having low risk of bias, but provided contradictory results, as follows:

- The European Randomized Study of Screening for Prostate Cancer (ERSPC) reported that screening led to a significant reduction in prostate cancer-specific mortality (RR 0.84; 95% CI 0.73 to 0.95). This study was the only RCT that reported a significant reduction in prostate cancer-specific mortality in a pre-specified subgroup of men aged 55 to 69 years of age.
- The US Prostate, Lung, Colorectal and Ovarian (PLCO) Cancer Screening Trial reported that screening did not lead to any significant benefit (RR 1.15; 95% CI 0.86 to 1.54).

A meta-analysis on these two studies with low risk of bias alone (ERSPC and PLCO) showed that there was no significant difference in prostate cancer-specific mortality (RR 0.96; 95% CI 0.70 to 1.30). Prostate cancer was diagnosed significantly more frequently among screened men than among unscreened men (RR 1.30; 95% CI 1.02 to 1.65). Localized prostate cancer was diagnosed more frequently among screened men (RR 1.79; 95% CI 1.19 to 2.70), and advanced prostate cancer was diagnosed less frequently among screened men (RR 0.80; 95% CI 0.73 to 0.87).

Screening led to a variety of harms, including bleeding, bruising, short-term anxiety, overdiagnosis and overtreatment, infection, blood loss, requirement of transfusion, pneumonia, erectile dysfunction and incontinence. The adverse events related to biopsies guided by transrectal ultrasound (TRUS) included infection, bleeding and pain. No deaths were directly related to any biopsy procedure. None of the RCTs provided any detail about quality of life as an outcome. The authors concluded that prostate cancer screening did not significantly decrease prostate cancer-specific mortality. A single RCT (ERSPC) found a 21% reduction of prostate cancer-specific mortality in a pre-specified subgroup of men aged 55 to 69 years. A meta-analysis showed that there was no significant reduction in prostate cancer-specific or overall mortality. Harm of moderate severity is frequently associated with prostate cancer-specific screening and further diagnostic evaluations. Overdiagnosis and overtreatment occur frequently and are associated with treatment-related harm. Individuals need to be aware of this when they are deciding whether to undergo screening. A reduction in prostate cancer-specific mortality may take up to 10 years to accrue and therefore men whose life expectancy is less than 10 to 15 years need to be aware that screening is unlikely to be beneficial. No studies examined the independent role of screening by means of digital rectal examination. For further details, refer to the original abstract, available at: http://onlinelibrary.wiley.com/doi/10.1002/14651858.CD004720.pub3/full.

Six systematic reviews that did not find any primary study that fulfilled their inclusion criteria (empty reviews) were also included in our review. Their aims were to evaluate the following:

- Screening using urinary dipsticks for reducing morbidity and mortality;^11^
- Mammography versus mammography plus ultrasonography for breast cancer screening testing in women at average risk;^13^
- Screening for nasopharyngeal cancer;^20^
- Screening for esophageal cancer;^21^
- Screening for testicular cancer;^24^
- Follow-up strategies after treatment (large loop excision of the transformation zone (LLETZ)) for cervical intraepithelial neoplasia (CIN): Impact of human papillomavirus (HPV) test.^25^

Thus, no recommendations in relation to the topics of these six systematic reviews can be made until primary studies, preferably RCTs with strong methodological quality, have been published.

## DISCUSSION

The present study included 17 Cochrane systematic reviews: 15 relating to screening for specific types of cancer and 2 to screening for cancer in general. However, 6 of the 17 reviews did not find any clinical trial that met the inclusion criteria, and therefore the authors of those “empty reviews” were unable to provide recommendations on the benefits and risks of screening. These last reviews were on screening for bladder, breast, nasopharyngeal, esophageal, testicular and cervical cancer.

Many clinical trials included in the systematic reviews were small, had short-term follow-up and were of poor methodological quality, which limited the quality of evidence available for many relevant outcomes. This was notable in relation to the reviews on screening among individuals with idiopathic deep venous thrombosis,^10^ screening for hepatocellular carcinoma among individuals with chronic hepatitis B^18^ and screening for oral cancer in the general population.^22^

Regarding the screening strategies most commonly used by professionals on populations, a systematic review^14^ showed that routinely performed breast self-examination did not reduce cancer mortality and also doubled the number of biopsies with benign outcomes.

There continues to be discussion concerning mammography for breast cancer screening. On the basis of a Cochrane systematic review, mammographic screening does not seem to have benefits regarding breast cancer mortality or cancer-related mortality after 10 and 13 years respectively.^15^

For prostate-specific antigen testing with or without digital rectal examination for prostate cancer screening, the results from the two major RCTs are inconsistent regarding the benefits relating to mortality.^23^

Regarding the implications of this study for clinical practice, the evidence found here does not support most routinely performed screening approaches, given their lack of clinical effectiveness. Patients need to be aware of the risks of false-positive and false-negative results before undergoing screening tests.

Concerning the implications of this study for further research, since only four systematic reviews provided high-quality bodies of evidence, there is a clear need for better designed and better conducted clinical trials for assessing the clinical outcomes of the various screening methods that are frequently used in clinical practice and for assessing the effectiveness of screening for the vast majority of cancers.

## CONCLUSION

This overview brought together 17 systematic reviews with distinct variations in the level of evidence presented, from low to high. The evidence found in this overview did not support most of the commonly used screening tests for cancer. Therefore, we take the view that patients need to be aware of, and well-informed about the possibilities of false positives and false negatives before undergoing such tests. The number of studies with high-quality evidence level pointing towards significantly decreased mortality was low. These studies addressed two issues: low-dose computed tomography for high-risk individuals, which seemed to reduce lung cancer mortality; and flexible sigmoidoscopy and fecal occult blood tests, which seemed to reduce colorectal cancer mortality.

Further studies with better quality are needed in order to assess the effectiveness of screening tests for cancer as well their side effects.
